# Identification and Functional Characterization of Three NoLS (Nucleolar Localisation Signals) Mutations of the CDC73 Gene

**DOI:** 10.1371/journal.pone.0082292

**Published:** 2013-12-05

**Authors:** Valerio Pazienza, Annamaria la Torre, Filomena Baorda, Michela Alfarano, Massimiliano Chetta, Lucia Anna Muscarella, Claudia Battista, Massimiliano Copetti, Dieter Kotzot, Klaus Kapelari, Dalia Al-Abdulrazzaq, Kusiel Perlman, Etienne Sochett, David E. C. Cole, Fabio Pellegrini, Lucie Canaff, Geoffrey N. Hendy, Leonardo D’Agruma, Leopoldo Zelante, Massimo Carella, Alfredo Scillitani, Vito Guarnieri

**Affiliations:** 1 Gastroenterology, IRCCS “Casa Sollievo della Sofferenza”, San Giovanni Rotondo (FG), Italy; 2 Laboratory of Oncology, IRCCS “Casa Sollievo della Sofferenza”, San Giovanni Rotondo (FG), Italy; 3 Medical Genetics, IRCCS “Casa Sollievo della Sofferenza”, San Giovanni Rotondo (FG), Italy; 4 Department of Molecular Biology, Molecular Stamping (Fondazione Bruno Kessler), Povo (TN), Italy; 5 Endocrinology, IRCCS “Casa Sollievo della Sofferenza”, San Giovanni Rotondo (FG), Italy; 6 Unit of Biostatistics, IRCCS “Casa Sollievo della Sofferenza”, San Giovanni Rotondo (FG), Italy; 7 Division of Human Genetics, Department of Medical Genetics, Molecular and Clinical Pharmacology, Innsbruck Medical University, Innsbruck, Austria; 8 Clinical Department of Pediatrics, Innsbruck Medical University, Innsbruck, Austria; 9 Division of Endocrinology, The Hospital for Sick Children, Toronto, Ontario, Canada; 10 Departments of Laboratory Medicine and Pathobiology, Medicine and Genetics, University of Toronto, Ontario, Canada; 11 Laboratory of Clinical Epidemiology of Diabetes and Chronic Diseases, Consorzio Mario Negri Sud, Santa Maria Imbaro (CH), Italy; 12 Calcium Research Laboratory and Hormones and Cancer Research Unit, Royal Victoria Hospital, Montreal, Quebec, Canada; University of Catania, Italy

## Abstract

Hyperparathyroidism Jaw-Tumour Syndrome (HPT-JT) is characterized by primary hyperparathyroidism (PHPT), maxillary/mandible ossifying fibromas and by parathyroid carcinoma in 15% of cases. Inactivating mutations of the tumour suppressor *CDC73/HRPT2* gene have been found in HPT-JT patients and also as genetic determinants of sporadic parathyroid carcinoma/atypical adenomas and, rarely, typical adenomas, in familial PHPT. Here we report the genetic and molecular analysis of the *CDC73/HRPT2* gene in three patients affected by PHPT due to atypical and typical parathyroid adenomas, in one case belonging to familial PHPT. Flag-tagged WT and mutant *CDC73/HRPT2* proteins were transiently transfected in HEK293 cells and functional assays were performed in order to investigate the effect of the variants on the whole protein expression, nuclear localization and cell overgrowth induction. We identified four *CDC73/HRPT2* gene mutations, three germline (c.679_680delAG, p.Val85_Val86del and p.Glu81_Pro84del), one somatic (p.Arg77Pro). In three cases the mutation was located within the Nucleolar Localisation Signals (NoLS). The three NoLS variants led to instability either of the corresponding mutated protein or mRNA or both. When transfected in HEK293 cells, NoLS mutated proteins mislocalized with a predeliction for cytoplasmic or nucleo-cytoplasmic localization and, finally, they resulted in overgrowth, consistent with a dominant negative interfering effect in the presence of the endogenous protein.

## Introduction

Hyperparathyroidism Jaw-Tumour Syndrome (HPT-JT) is characterized by primary hyperparathyroidism (PHPT) (due to parathyroid carcinoma in 15% of cases), maxillary and mandible ossifying fibromas [1,2], renal and uterine tumours [3,4]. Inactivating mutations of the *CDC73*/*HRPT2* (henceforth *CDC73*) gene, encoding the 531 amino acid nuclear parafibromin protein, have been found in HPT-JT patients [5], while biallelic inactivation of this gene may be seen in sporadic parathyroid carcinoma [6], consistent with a tumour suppressor function. *CDC73* gene inactivating mutations are also associated with other neoplasia such as clear cell, papillary, chromophobe renal cell carcinomas, oncocytomas, Wilms tumour [7] and more rarely biliary duct carcinoma [8]. Parafibromin is a component of the PAF1 transcription complex [9] associating with RNA polymerase II to regulate several processes, from initiation of transcription to mRNA maturation, by associating with mRNA processing and polyadenylation factors [10]. Like its Drosophila homologue, Hyrax, human parafibromin interacts physically with the β-catenin protein via its conserved N-terminal sequence, suggesting a role in the regulation of WNT pathway targeted genes [11]. Being the parafibromin mainly a nuclear protein, it possesses different nuclear and nucleolar localisation signals (NLS and NoLS, respectively) that have been functionally investigated previously [12,13,14].

Inactivation of *CDC73* gene occurs frequently by frameshift or non-sense mutations while missense mutations are rare [15]. Among the naturally missense mutations, so far, only two variants were identified in the three NoLS: the p.Arg91Pro [16] and the p.Leu95Pro [17], located within or close to the NoLS 76-92, respectively. Only p.Leu95Pro has been functionally characterized, showing that the mutated protein localizes to the nucleus, but fails to localize to the nucleoli [18].

Here we report the identification and the functional characterization of three different *CDC73* mutations located within NoLS 76-92, found in three subjects affected by PHPT due to parathyroid atypical adenoma or typical adenoma, the latter belonging to familial PHPT. These variants gave us the opportunity to explore the outcome of naturally-occurring missense mutations of the NoLS sequence about the parafibromin protein expression, function and localization.

## Patients

### Case I

At the age of 12 years (in 2001) the patient was referred to the Orthopedic Clinic (Town) due to persistent pain in the left ankle following a trauma. Conventional x-ray of the left lower extremity revealed a lesion in the distal tibia which was diagnosed as a “non-ossifying fibroma”. Two years later, the patient underwent distal supracondylar osteotomy at the Dept of Orthopedics of the Innsbruck Medical University, Austria, because of valgus deformity of the left femur. Postoperative course was complicated by polydipsia and vomiting and laboratory measurements revealed marked hypercalcemia and elevated parathyroid hormone. Clinical chemistry profile showed: albumin adjusted serum Ca 17.2 mg/dL (normal range 8.4-10.2), PTH: 571.8 pg/mL (10-72); calcitonin, gastrin and urinary catecholamines were normal. Unilateral exploration of the neck with resection of an adenoma of the left inferior parathyroid gland was performed. The pathological diagnosis was of parathyroid adenoma (Table 1).

**Table 1:** Clinical, genetic and histology data of the probands.

	Case I	Case II	Case III
Gender	m	m	m
Age at diagnosis	12	17	57
Ca_alb-adj_	17,2	14,32	14,82
PTH	571	2164	1328
P	NA	0,77	1,82
Alk Phosp	NA	2744	1543
Hystology	typical adenoma	atypical adenoma	atypical adenoma
CDC73 (germ)	c.252_257del6	c.242_253del12	c.679_680delAG
Somatic	NA	NA	c.231CG
Familiarity	Y	Y	NA

It is noteworthy that the father of the patient had been operated on for the removal of a parathyroid adenoma at 30 years of age, and that two aunts from the father’s side had been diagnosed with parathyroid adenomas. In May 2008 the biochemical follow up of the available relatives identified, in the brother, high albumin adjusted serum calcium (11.36 mg/dL, range 8.4-10.2) and PTH (103.8 pg/mL, range 10-72) and in December 2008 a parathyroid adenoma was removed.

### Case II

The patient, a 17 year old boy, who had suffered for the past two years for a long standing generalized bone pain, was admitted at the Hospital for Sick Children in Toronto after a 3 day history of bilateral sudden acute hip pain. Clinical chemistry profile was: albumin adjusted serum Ca 14.32 mg/dL (range 8.4-10.2), phosphate 0.77 mg/dL (2.7-4.5), alkaline phosphatase 2744 U/L (up to 270), and PTH 2164 pg/mL (10-72, Table 1). Neck ultrasonography and parathyroid radionuclide scan identified a right inferior parathyroid lesion of 1.6 x 1.8 x 2.1 cm that was removed. Histological report was of atypical adenoma without vascular or capsular invasion. Family history included the presence of multiple uterine fibroids and a mixed epithelial and stromal tumor of the right kidney in the mother.

### Case III

The patient was healthy up to 57 years. Since then he suffered of a progressive asthenia and generalized bone pain. He was admitted at the Endocrine Unit of the IRCCS Casa Sollievo della Sofferenza Hospital in San Giovanni Rotondo (Italy) and the clinical chemistry profile was: albumin adjusted serum Ca 14.82 mg/dL (normal range 8.4-10.2), phosphate 1.82 mg/dL, (2.7-4.5), creatinine 1.04 mg/dL (0.6-1.1), alkaline phosphatase 1543 U/L (up to a 270), PTH 1328 pg/mL (10-72), urinary Ca 435 mg/day (100-250, Table 1). Serum gastrin, calcitonin and PRL, urinary 5-hydroxyl indolacetic acid and catecolamines were normal . Neck ultrasonography revealed a nodule of 18 x 14 x 25 mm on the postero-lateral side of the right thyroid lobe. Kidney and urinary tract urography showed microcalcifications. He underwent surgical intervention of “en bloc” parathyroidectomy and ipsilateral hemithyroidectomy with removal of a histologically diagnosed parathyroid atypical adenoma.

## Materials and Methods

### Molecular Screening

All the patients or their parents/relatives (if the patient was underage or deceased) gave informed signed consent and the study was approved by the Ethical Committee at “Casa Sollievo della Sofferenza” Hospital. For Cases I and II, peripheral blood was collected, but no tumour tissues were available at the time of the study. For Case III, we collected the tumour tissue and matched normal non-tumoral tissue. For all the subjects, molecular screening of the whole *CDC73* coding sequence (17 exons, including exon-intron boundaries) was performed by PCR amplification and direct sequencing as described [19]. Mutations were confirmed by sequencing in both directions with forward and reverse primers on the original amplicon and on a different PCR product.

### cDNA expression vectors

All the variants were introduced in a Myc-Flag tagged human *CDC73* cDNA expressing pCMV6 vector (Origene). Briefly, mutagenesis reactions were conducted in a total volume of 50 uL containing 100 ng DNA, 5 ul 10X buffer, 125 pmol (final) of each primer [p.Arg77Pro, For: 5'- ctgtttatgtccgacCtgcagctgtaagtag-3’ and Rev: 5’-ctacttacagctgcaGgtcggacataaacag 3’, mutated bases are in capital; p.Val85_Val86del, For: 5’- gctactgaaaatattcctagaagacctgatcgaa-3’ and Rev: 5’- ttcgatcaggtcttctaggaatattttcagtagc-3’; p.Glu81_Pro84del, For: 5’-tgtccgacgtgcagctactgtggttagaagacctgatcga-3’ and Rev: 5’- tcgatcaggtcttctaaccacagtagctgcacgtcggaca-3’], 8 uMol (final) dNTPs and 1U Pfu Taq polymerase (Promega). PCR conditions were: 95°C x 2 min, and 18 cycles of 95°C x 30 sec, 55°C x 1 min and 72 °C X 12 min. One microlitre of Dpn1 (New England Biolabs) was added to digest parental DNA and 3 uL used to transform DH5a cells (Invitrogen). Colony PCR and sequencing identified the mutated clones. Midipreps were performed with Plasmid Midi Kit (QIAGEN).

### Cell culture

Human embryonic kidney (HEK293) cells (ECACC) were cultured in DMEM/Ham’s F12 (BioSpa) supplemented with 10% Fetal Bovine Serum (BioSpa) and 1% penicillin/streptomycin (BioSpa) and incubated at 37°C in a humidified, 5% CO2 incubator. Wild-type and mutant vectors were transfected in duplicate or quintuplicate (in case of the MG132 or cycloheximide and overgrowth assays, respectively) using Lipofectamine 2000 Transfection reagent (Invitrogen) following the manifacturer’s instructions. For each experiments HEK293 cells were used with a number of passages < 10.

### Western blot

Total cell proteins were extracted in RIPA buffer (150 mM NaCl, 50 mM Tris-HCl, 1% Nonidet P-40, 0.1% sodium dodecyl sulfate, 0.5% sodium deoxycholate, pH 8.0) and 50 ug of proteins were loaded onto a 12% SDS polyacrylamide gel. Proteins were electrotransferred to PVDF membrane (Millipore, Billerica, MA), blotted overnight at 4°C with rabbit anti Flag monoclonal antibody (Cell Signaling Technology) and for 1 h at room temperature with the horseradish peroxidase-conjugated goat anti-rabbit IgG antibody (Biorad) as secondary antibody. Membranes were stripped with Re-Blot Solution (Millipore) and β-tubulin rabbit monoclonal antibody (Cell Signaling Technology) was blotted as loading control. For quantitative measurement, films were scanned by densitometry and the spots corresponding to proteins were analysed by using the ImageJ-National Institutes of Health image-processing program (http://rsbweb.nih.gov/ij/).

### Protein degradation assessment

After 48 hours from the transfection, MG132 (Sigma Aldrich, 25 μM final concentration) was added to one set of cells and protein extraction performed 4 hours later. In order to determine the steady-state level of the mutated proteins, after 48 hours from the transfection, cycloheximide was added (BioMol, 500 μM final concentration) and, at different time points (0, 1, 2, 4, 8 h), total cell protein extraction was carried out and western blots performed.

### Real-Time quantitative Polymerase Chain Reaction (RT-qPCR)

HEK293 cells were cultured in 6 well plates and transfected with WT and mutants vectors as previously described. After 48 h, cycloheximide (BioMol, 500 μM final concentration) was added. 4 hours later, total RNA was extracted using the Invitrogen TRIzol reagent (Carlsbad, CA) [20] and concentrations were quantified by Nanodrop spectrophotometer. 500 ng of total RNA was subjected to reverse transcription using the High Capacity cDNA Reverse Transcription kit (Applied Biosystems). 

Fluorescence based real time quantitative PCR (RT-qPCR) was set up in 384-well plates in a total volume of 10 μl containing 1 μL cDNA, 0,5 μL PrimeTime qPCR Assay (IDT) specifically designed on the Myc-Flag sequence (primers and probe sequences are available from the Authors) and 5 μL of PCR Master Mix (Applied Biosystems). Reactions were run on ABI PRISM 7900 Sequence Detection System (Applied Biosystems). Transcription levels of Flag-tagged *CDC73* WT and mutated transcripts were normalized using the expression level of the human large ribosomal protein, *RPLPO* (IDT) as reference gene. A relative quantification method with standard curve was developed, mRNA levels in each sample were determined as the ratio of the *CDC73* expression level to the RPLPO expression [21].

### Proliferation assay

Twenty thousands cells were seeded in 96 well plates: WT and mutants vectors were transfected in fifteen replicates (five replicates for each time) with Lipofectamine 2000 (Invitrogen) as previously described and at definite time points (24, 48 and 72 hours), 10 µL of MTT Reagent (Roche) was added. After 4 hours the medium was removed and ice-cold isopropanol added. Then the absorbance at 450/620 nm was measured with an Elisa reader. 

### Indirect immunofluorescence (IFL)

HEK293 cells were grown and transfected on coverslips with WT and mutant vectors as previously described. After 48 h they were fixed for 30 min in 4% paraformaldehyde at 4 °C. Coverslips were then washed three times in PBS 1 X and incubated with the rabbit anti Flag antibody (Cell Signaling Technology) in PBS 1X, bovine serum albumin (BSA) 1X and Triton X-100 1X for 2 h at room temperature (RT). After three washes in PBS 1X, cells were incubated 2 h at room temperature with FITC-labeled anti-rabbit antibody (Li StarFish). After rinsing three times in PBS 1X, coverslips were mounted on microscope slides. For fluorescence microscopy, slides were mounted for immunofluorescence on glass slides with Vectashield (Vector Laboratories, Burlingame, CA) containing 4,6- diamidino-2-phenylindole (DAPI) and observed using a Nikon Eclipse E600 microscope.

### 3-D mutation prediction

To investigate the possible structural consequences of the identified variants, the 3-dimensional structure of the WT and mutant CDC73 proteins were inferred using the Phyre2, Protein Homology Fold RecognitionEngine, [22], server created by the Structural Bioinformatics Group, Imperial College, London. The *.pdb model generated from Phyre2 was loaded and the 3-D structure was visualized using the ChemDraw software (Cambridge Software, http://www.cambridgesoft.com, last access: july 2013).

### Statistical analysis

Experiments were repeated three times and representative experiments are shown. Results are expressed as means ± standard errors (SE). Group comparisons were performed using Student’s t-test. A p-value less than 0.05 was considered statistically significant. All analyses were performed using SAS Release 9.1 (SAS Institute, Cary, NC, USA). 

## Results

### Molecular Analysis Of CDC73 Gene

**Figure 1:**
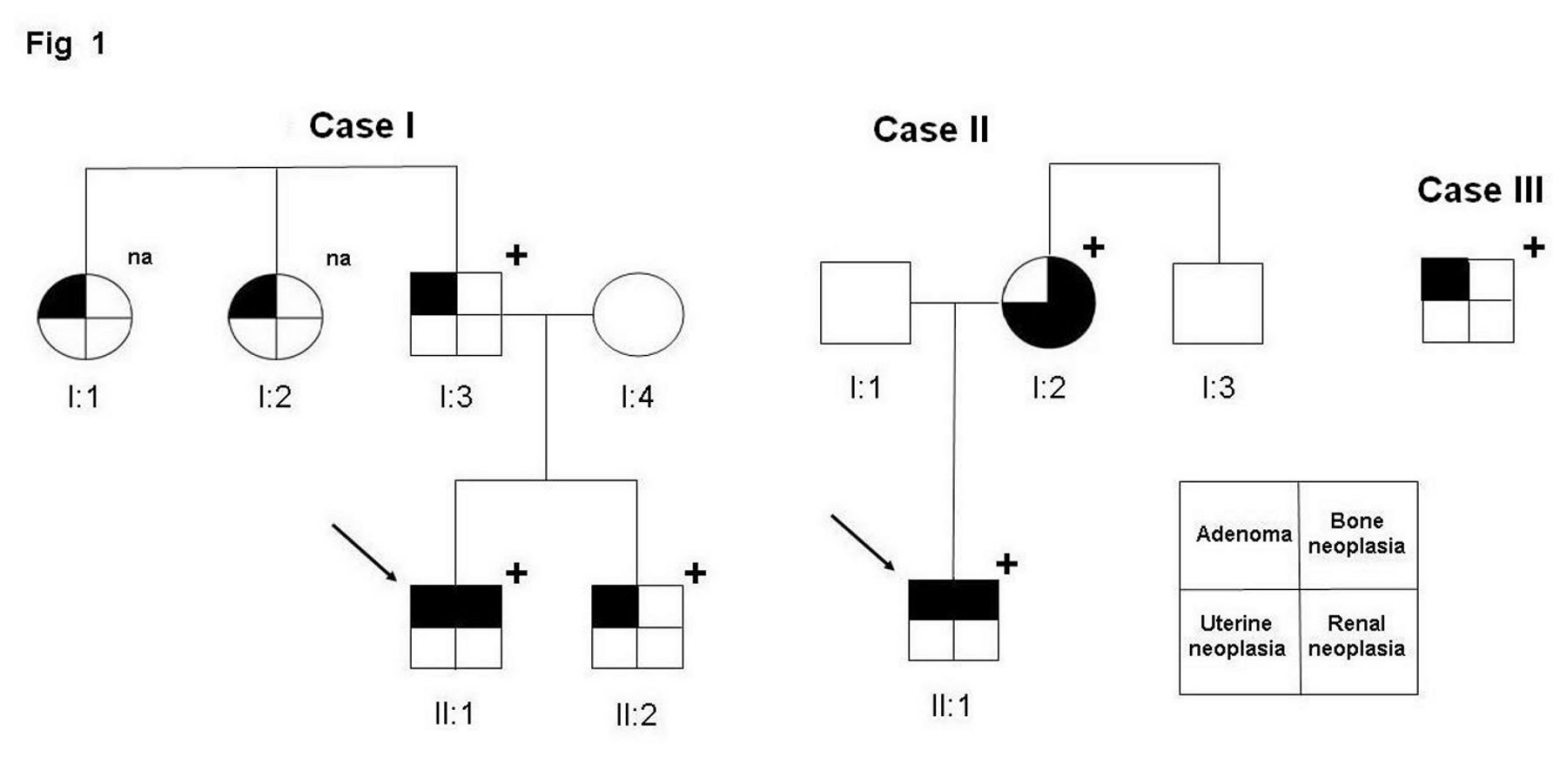
Family trees for the three Cases. The arrow indicates the proband; “+” indicates the presence of the mutation; na = DNA not available. For Case III no relatives were recruited at the time of this report.

Case I. The analysis on DNA from blood leukocytes reported a novel constitutional in-frame deletion of two valines in the exon 3, namely c.252_257del6 (p.Val85_Val86del, hereafter delVV). The analyses extended to the proband’s brother and father identified the same mutation in both relatives. DNA from the two paternal aunts was not available at the time of report (Figure 1).

Case II. The screening of DNA extracted from blood leukocytes identified a novel in-frame constitutional deletion of 4 amino acids in exon 3, c.242_253del12 (p.Glu81_Pro84del; hereafter abbreviated delENIP, from the one-letter code of the deleted residues). Analysis extended to the relatives revealed the same mutation in the mother and the maternal uncle. (Figure 1).

Case III. The molecular screening identified a biallelic inactivation of the *CDC73* gene, consisting of: a previously reported [5] frameshift deletion of the exon 7, namely c.679_680delAG (R227fs*263), found on DNA extracted from non tumoral tissue and a missense variant of the exon 2, c.231CG (p.Arg77Pro, hereafter R77P) found in the tumour tissue [19]. Family studies were attempted but samples were not available at the time of report (Figure 1).

### Conservation of mutated residues in the phylogenetic three

**Figure 2:**
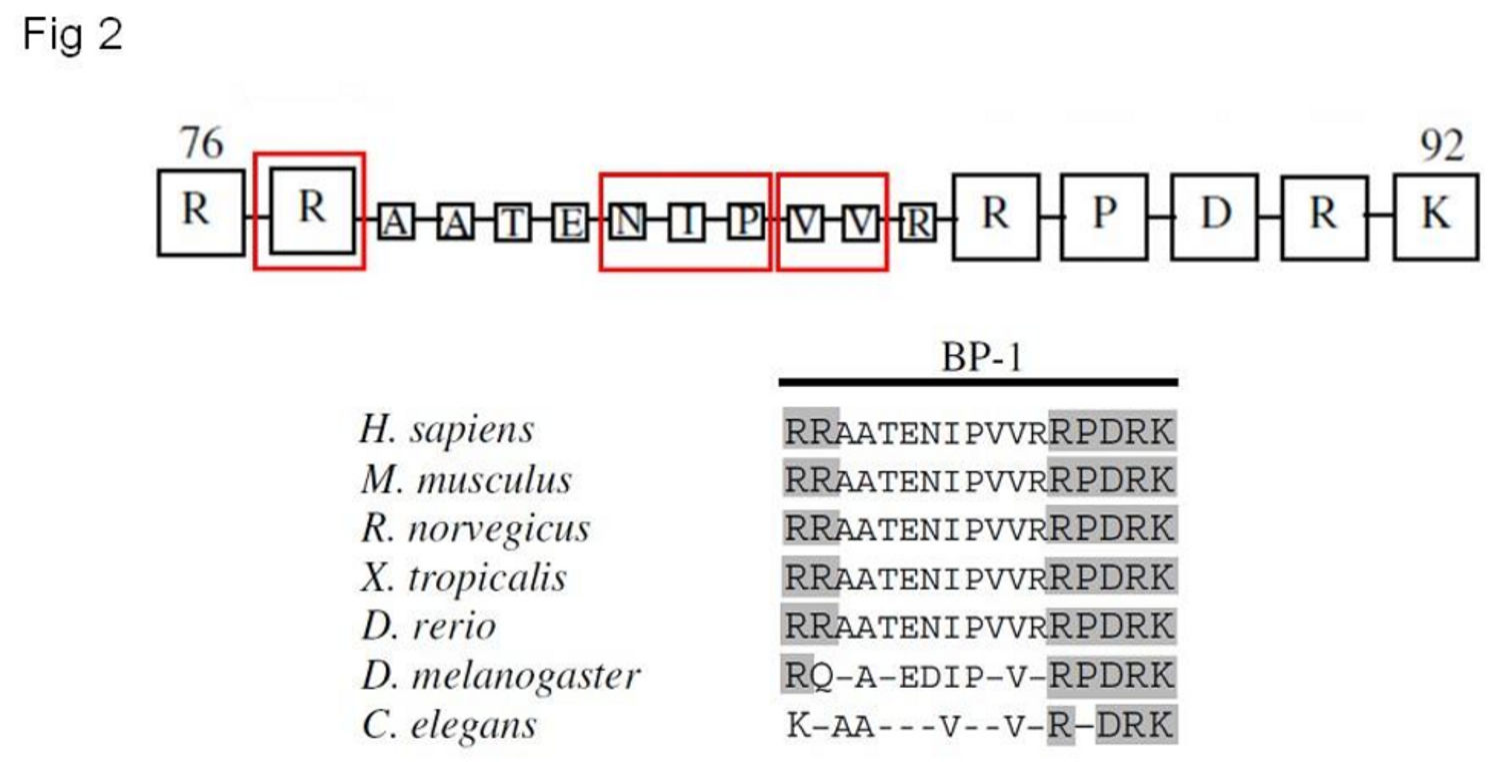
Phylogenetic analysis. Multialignment of parafibromin protein sequences and phylogenetic comparison shows the high conservation of residues of NoLS 76-92 motif. In red blocks the three variants here reported (modified by ref.13).

Three variants out of four were located in the NoLS 76-92 signal: the analysis of the evolutionary conservation showed that all the residues are conserved in the vertebrate phylogenetic tree. While the R77 and all the ENIP residues are present in fishes, only one of the two valines (the V85) is conserved in worms. This finding is additional evidence of the importance of these residues and prompted us to investigate more deeply the functional consequences of these mutations (Figure 2).

### CDC73 WT and mutant proteins expression

**Figure 3:**
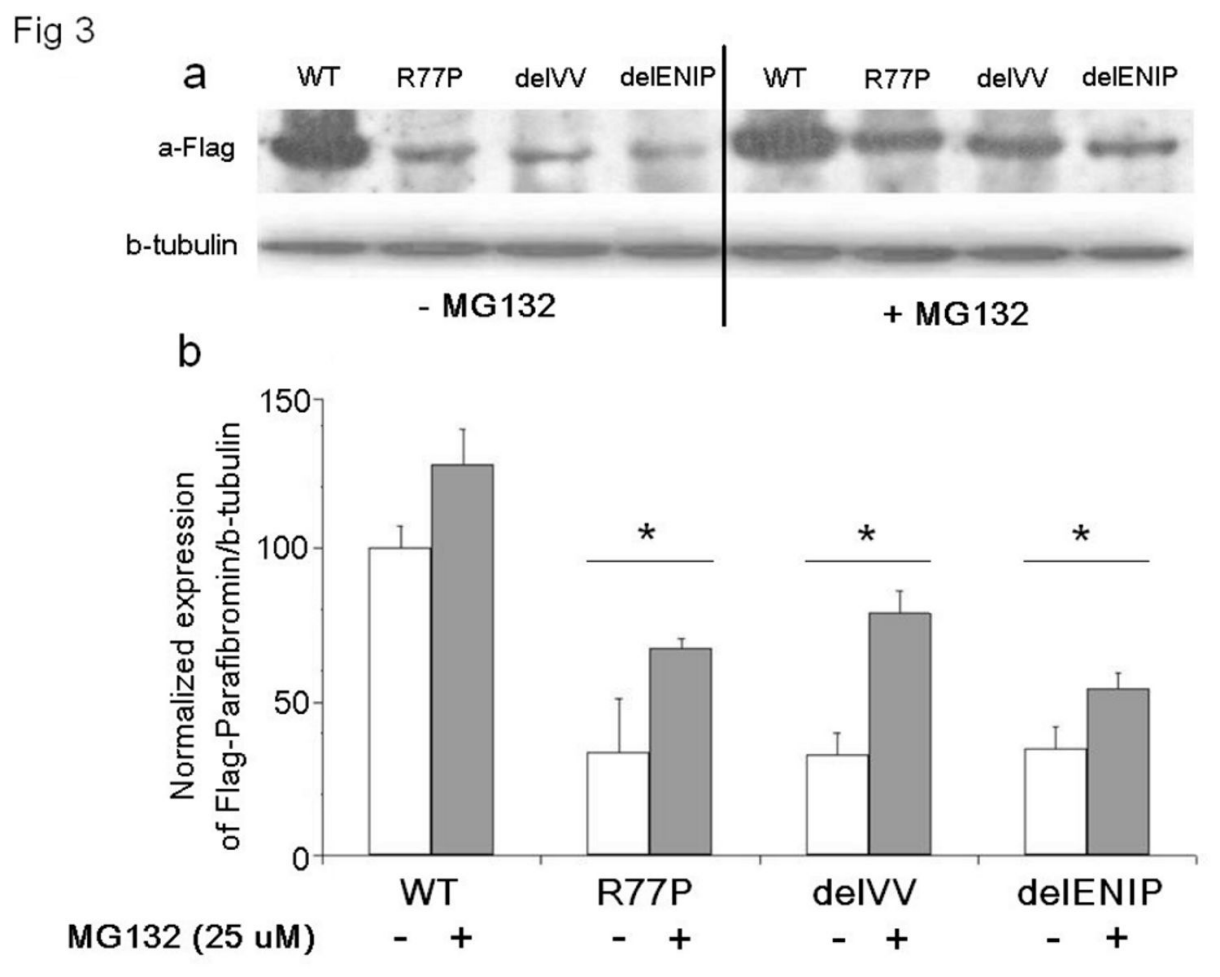
Immunoblot detection of CDC73. Total protein cell lysates from cell transfected with WT and mutants (R77P, delVV, delENIP) vectors were detected with Anti-Flag antibody showing that the mutant proteins are poorly expressed with respect to the WT: the same assay performed in presence of the proteasome inhibitor, MG132 (25 μM, final concentration), led to the partial recovery of the mutant proteins.

In order to understand the effect of the mutation on expression of the protein, an immunoblot detection was performed on HEK293 cells transiently transfected with Flag-tagged WT, R77P, delVV and delENIP expressing vectors. Mutated proteins were expressed by less than 35% of WT (Figure 3a). After treatment with a proteasome inhibitor, MG132 (25 μM final concentration), a partial rescue of the expression, up to the 60% (with respect to the untreated cells, Figure 3b), was observed.

### Quantitative RT-PCR of mutant mRNAs transcription levels

**Figure 4:**
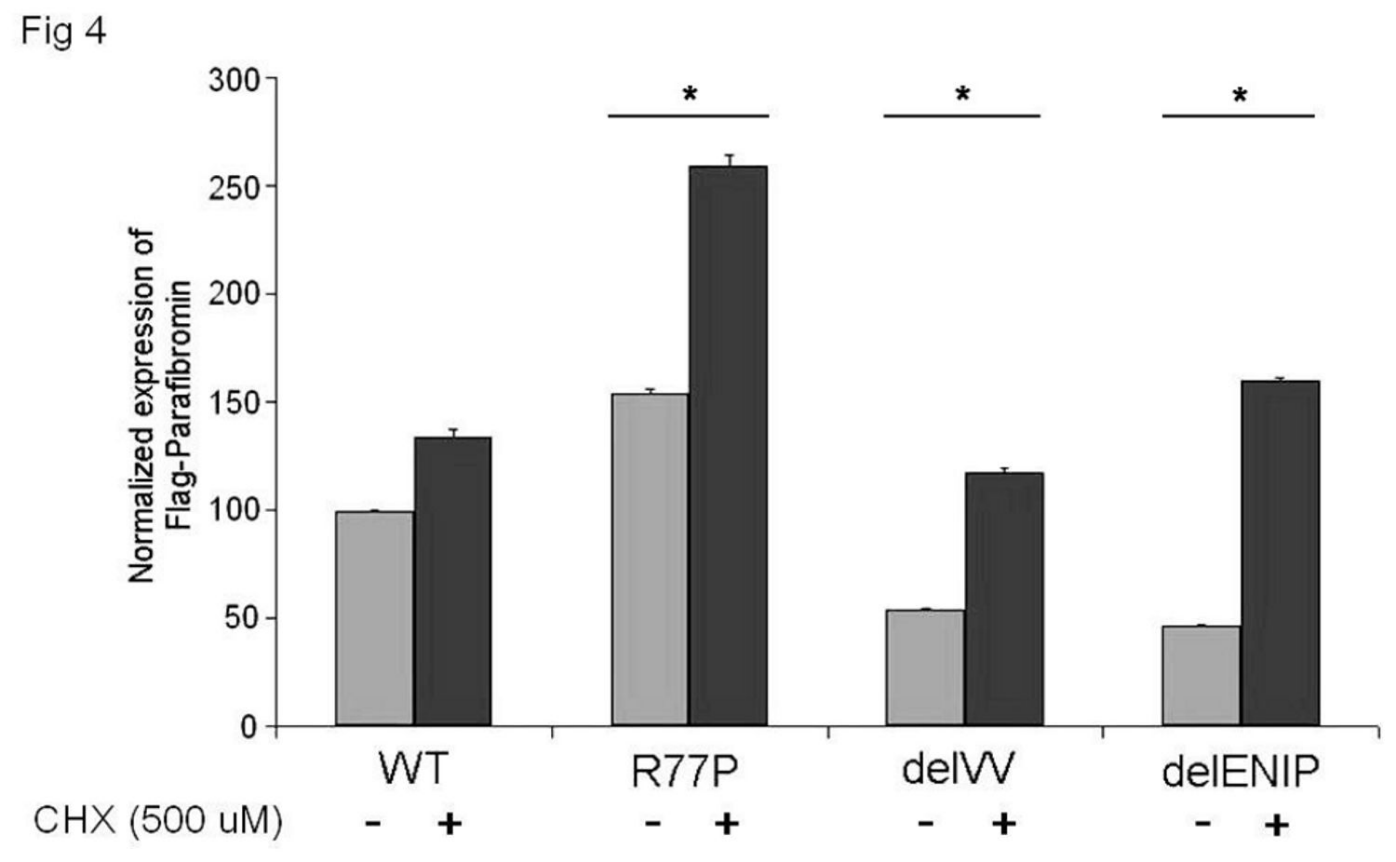
qRT-PCR expression of Flag parafibromin WT and mutant mRNAs. The assay, performed in presence and absence of cycloheximide (CHX 500 μM, final concentration) shows that the inhibition of the degradation process led to the total recovery of the mutant mRNAs.

The possibility of increased post transcriptional degradation was investigated by performing RT-qPCR in the presence/absence of cycloheximide. Figure 4 showed different results with regard the expression level of mutated mRNAs: while the mRNA carrying the R77P mutation was over expressed relative to WT, the other two mRNAs were underexpressed (40% less on average). Note that expression of all three mutant mRNAs increased in the presence of the inhibitor (Figure 4). 

### Half-life protein determination by CHX chase assay

**Figure 5:**
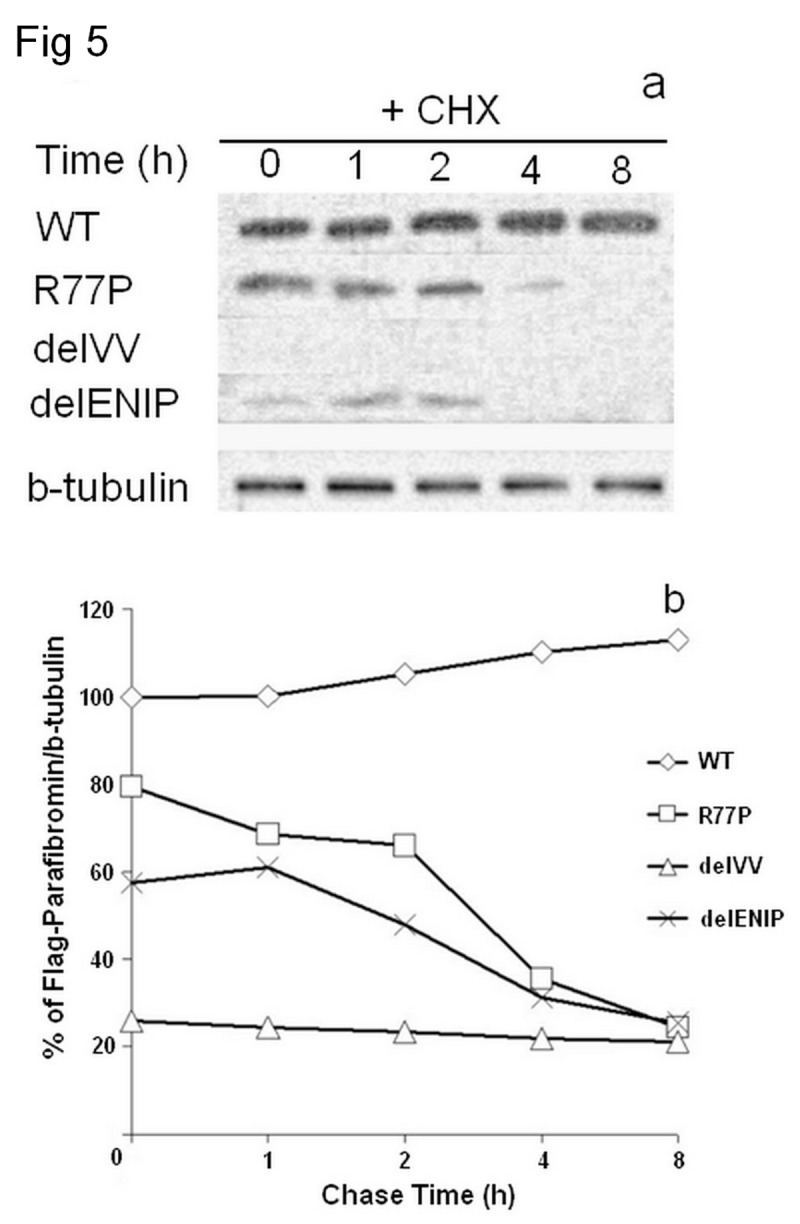
CHX Chase assay. The western blot performed in presence of MG132, after 48 h from transfection showed that the mutated proteins were degraded (a) up to the 80% with respect to the WT, with short half-lives around the 2,5 h (b).

To investigate about the steady state levels of the mutated proteins a CHX chase assay was performed. As shown in Figure 5, after 4 h from the drug administration (52 h after the transfection) the mutant proteins were underexpressed by 20% (R77P) to 80% (delVV) compared with the WT, depending on the mutation, suggesting that all three mutated proteins are relatively instable.

### Immunofluorescence detection of WT and mutated proteins

**Figure 6:**
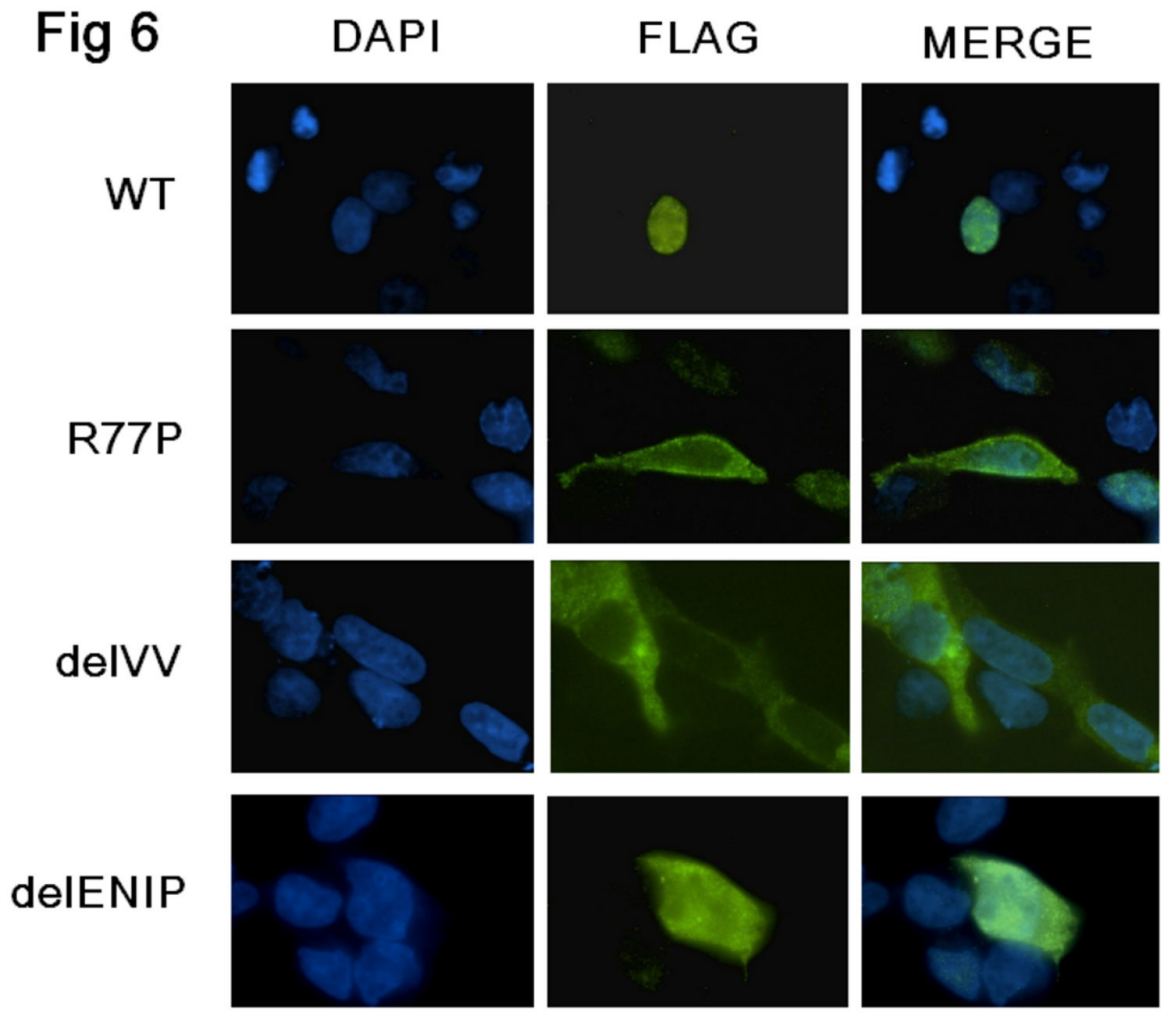
Immunofluorescence. Microscopic images of HEK293 cells transfected with Flag parafibromin WT and the three different mutants vectors, analyzed for nuclear staining and protein localization using the anti-Flag antibody. The WT protein localized into the nucleus, the R77P and the delVV mutant proteins localized into the cytoplasm while the delENIP mutant protein localised into both cytoplasm and nucleus.

The IFL explored the localization of the CDC73 wild type and different mutated proteins. With regard to the R77P and the delVV variants, a definite cytoplasmatic localization without any detectable nuclear expression was observed. With regard to the delENIP protein, while it localized within the cytoplasm, it retained the ability to traffick into the nucleus (Figure 6).

### Cell proliferation assay

**Figure 7:**
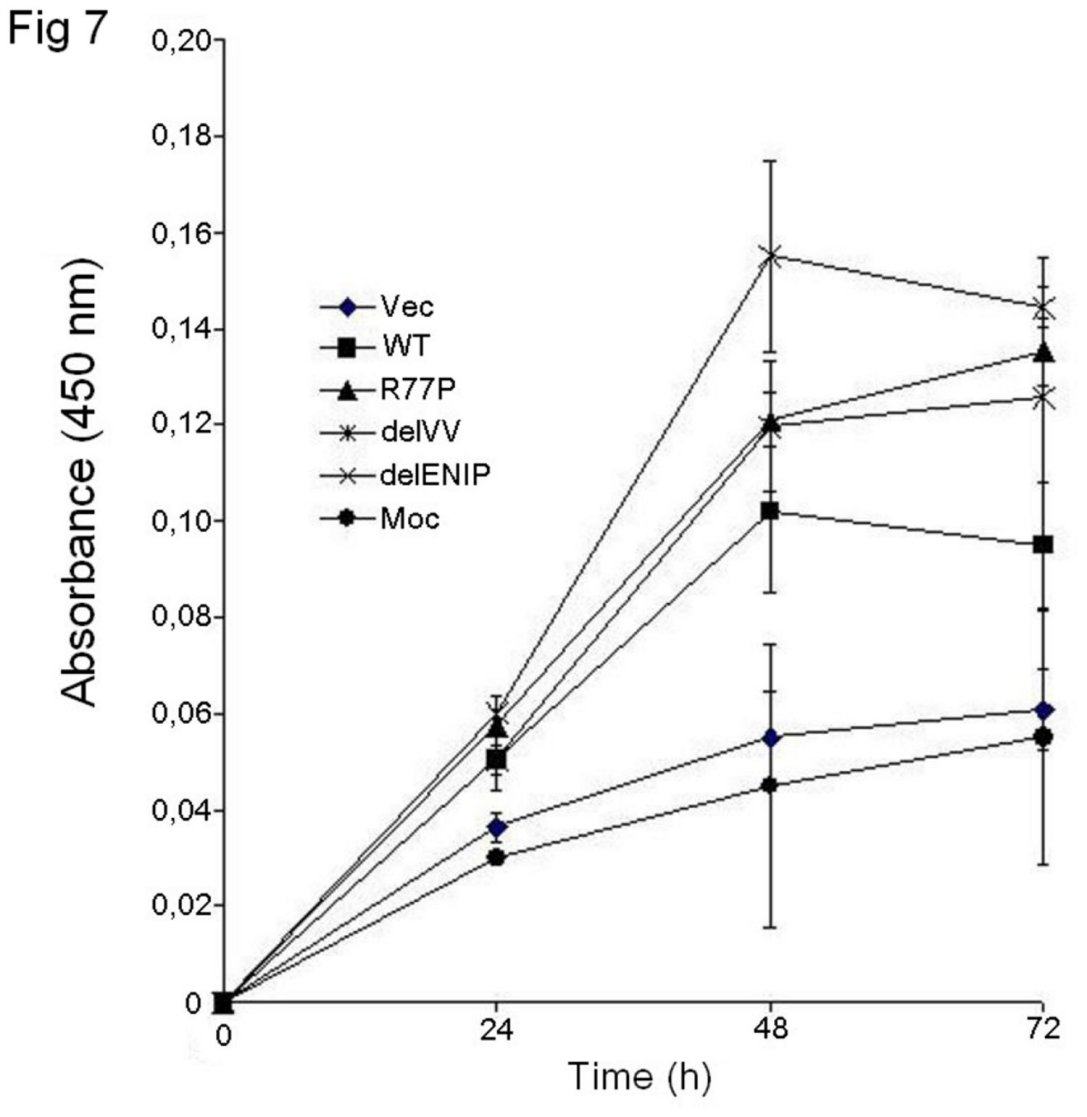
MTT test. Proliferation assay at different time points (24, 48 and 72 h). The WT vector limited the cell growth, while all the three mutant vectors caused cell overgrowth in presence of the endogenous parafibromin, suggesting a dominant interfering effect.

We then sought to look into the effect of the mutated proteins on cell proliferation. The assay carried out at three different time points (Figure 7) showed that the WT vector limited the cell growth while mutated vectors induced higher cell proliferation rate, taking also into account the expression of the endogenous parafibromin. 

### NoLS mutations: 3-D prediction

**Figure 8:**
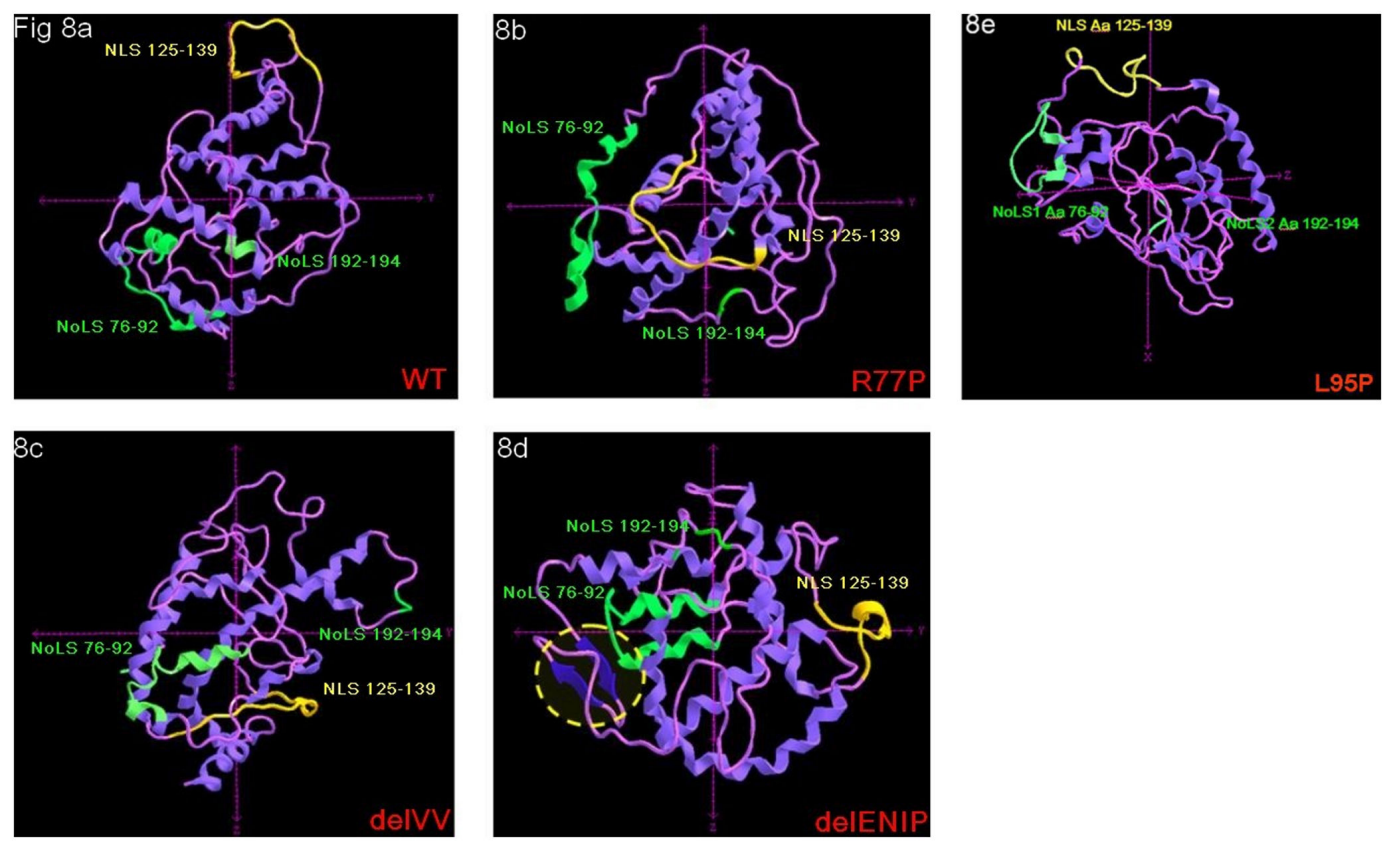
3-D modelling prediction. The different localization and folding level of the NLS 125-139, NoLS 76-92 and NoLS 192-194 are shown; a: WT protein, the NLS 125-139 and the NoLS 76-92 locate at the outer of the protein; b and c: for the R77P and delVV mutant proteins, while the NoLS 76-92 do not show steric hindrance, the NLS 125-139 appears located within the protein structure; d: in case of the delENIP mutant the NLS 125-139 appeared strongly misfolded while two novel antiparallel β sheet fragments (Aa 15-27) are shaped and seem to contribute to the general inaccessibility of the NLS; e: the L95P mutation seems not to alter the either the conformation nor the accessibility of the NLS, as for the WT sequence: note that only for this model, axys are differently orientated to facilitate the observation of the protein motifs.

Comparison of mutants with wild-type using a 3-D modelling approach offered further insight. In the WT protein, the NLS 125-139 sequence appears to be protruding, thus more likely available for binding with other recognition proteins (Figure 8a). By contrast, in the model of the R77P and delVV mutants, the NLS 125-139 sequence was invaginated into the inner of the protein. This is compatible with a steric hindrance mechanism (Figure 8b and 8c). In the delENIP mutant model, the protein is strongly misfolded, both at the level of the NLS 125-139 sequence itself and because of the formation of two novel antiparallel β sheet fragments in the N-terminal tail (Aa 15-27, Figure 8d).

## Discussion

The tumor suppressor *CDC73* gene has been identified as the main genetic determinant of the HPT-JT syndrome and parathyroid carcinoma. So far, more than 120 different coding inactivating mutations and different genomic deletions have been detected in this gene [15,23-31]. The vast majority of coding alterations are frameshift and nonsense, predicting a truncated protein. Only a few missense mutations (11 out of 124) have been reported and, among those, only two - p.Arg91Pro [16] and p.Leu95Pro [17] - were associated with NoLS motifs [18].

We identified 4 *CDC73* gene mutations in 3 subjects affected by PHPT: 3 were germline, 1 was somatic. Three mutations were novel and fell into the NoLS and here we report their functional characterization.

The three NoLS variants, one missense (R77P) and two in-frame deletions (delVV and delENIP), are expected to leave the primary peptide sequence unaltered and in turn retain expression of the protein, unlike nonsense and frameshift mutations. However, immunoblot detection showed that these variants affect the protein expression with a reduction of up to 85%. Secondly, we looked for possible disruption in the steady-state level of the mutated protein due to the identified NoLS variants, as reported for other tumor suppressor genes [32]. The CHX assay demonstrated that the three NoLS mutations are short-lived compared to wild-type, and thus highly instable. After 48 h they were degraded by about 20% (for the R77P), 40% for the delENIP, and up to the 80% for delVV (with respect to the WT). This is consistent with the notion that an early proteolytic event is involved. 

Since we observed that proteasome inhibitor MG132 treatment was able to partially recover the CDC73 mutated protein (up to 80% with respect to untreated cells), we then sought if a concurrent pre-translational degradation process was also active. The RTqPCR assay, performed in presence of cycloheximide, offered a different picture. Two variants (delVV and delENIP) caused a strong reduction of the expression of corresponding mRNA (up to 54%) that was restored in presence of the translation inhibitor, suggesting that the increased instability of the mutated mRNAs might contribute to the whole loss of mutated protein products. With regard to the R77P variant, the mutated transcript was more expressed (up to 50%) than the WT: these contrary results, which in keeping with an earlier report [18] deserve further study. 

In keeping with the tumor suppressor gene hypothesis, parafibromin mutations have been reported to induce cell growth and proliferation [14,18,33,34]. We then verified if the NoLS variants acted in the same way. The MTT assay showed that the mutations affect the cell proliferation even in presence of the endogenous protein, while, as expected, the WT form showed a slower proliferation. These findings confirm previous observations about a possible dominant-negative interfering effect of the mutated parafibromin [18].

Bipartite nuclear/nucleolar localisation signals usually consist of two clusters of 2–3 positively charged basic residues separated by a 9–12 intermediate region containing proline [13]. In the parafibromin three bipartite NoLSs have been identified and characterized, the NoLS1 encompassing residues from 76 to 92 [12,13,35]. Among these 17 aminoacids, the basic residues (Arg76-77 and Arg88-Lys92) have been proved to be essential for the function of the signal [12]. The NoLS 76-92 seems not to play a pivotal role in trafficking the protein into the nucleus, rather than into the nucleolus, thus the different classification in NoLS and NLS - Nuclear Localisation Signals, NLS1 125-139 and NLS2 393-409 [12,35]. Moreover in absence of alterations of the primary NLS (125-139), mutations of NoLS 76-92 would not impair the nuclear localization of the parafibromin [35].

At the variance with these previous results but in keeping with others [12], we observed that, of our three NoLS proteins, two (R77P and delVV) showed almost negligible nuclear expression. In the third case (delENIP) the protein localized to both the cytoplasm and nucleus. Due to the low number of naturally occurring NoLS mutations so far identified and/or functional studies reported, we can not exclude that a role of other co-factors in this mis-localization. As suggested by the 3-D modelling approach, the NoLS mutations could induce a critical protein misfolding with a consequently inaccessibility of NLS 125-139 and the likely final impairing of binding with other co-factors (such as chaperonins and importins) assigned to the correct trafficking of nuclear proteins. In line with this hypothesis we predicted, in the same way, the NLS and NoLS conformation when the previously identified L95P mutation was introduced: the leucine-to-proline change seems to alter the folding of the protein, but in keeping with the reported nuclear localization of the mutated parafibromin, it seems not to influence the conformation nor the accessibility of the NLS sequence which remains at the outer surface of the protein structure.

The loss of function associated with *CDC73* gene mutations is often coupled to the inability of parafibromin to bind the PAF1 complex and the RNA polymerase II. The binding between mutated parafibromin with these complexes and/or their co-factors, such as Leo1 and CTR9 (Paf1/RNA polymerase II complex component) has been investigated in other cases [9,18,33]. However, the *CDC73* protein binding domain interacting with the PAFI complex is located at residues 226-413 [12], far from the mutation sites reported here and it is unlikely that any variant at the positions 77-84 could interfere with a protein domain located more than 150 residues downstream. Thus in our cases we presumed that this binding domain was not influenced by the NoLS mutation. However as reported above, our NoLS mutated proteins were almost totally degraded and if not degraded, they mislocalized in 2 cases out of 3 (R77P and delVV) while the PAFI and their co-factors are nuclear, thus with no supposed interaction. In the third case (delENIP) the protein localized partially into the nucleus and we can not exclude that a correct interaction with the above mentioned complexes could occur (as already reported) [18,35]. Nevertheless we believe that the main effect of these mutations resides in the high instability and, consequently, in the strong reduced expression of the mutated proteins.

The different sensitization at the proteasome degradation pathway of missense mutations has been reported for other tumor suppressor genes [32]. Our results are consistent with the hypothesis that proteolytic degradation is a relatively common mechanism for loss of function of tumor suppressor genes, at the same time giving rise to the possibility of new therapeutic interventions as proposed for other diseases [32].

In conclusion our work shed light on the outcome of novel mutations of the NoLS 76-92 of CDC73 gene. We found that the mutated proteins were highly instable and subjected to a critical transcriptional and translational degradation processes. Particularly the possibility that a pre-translational degradation may be responsible of loss of function of these mutated proteins will require further investigation. Moreover protein misfolding of the NoLS sequence might cause a dramatic displacement of the primary NLS 125-139, thus leading to a cytoplasmic mislocalisation of residual mutated protein product.
